# Catarrhal Diseases of the Upper Air-Passages

**Published:** 1904-04

**Authors:** 


### CATARRHAL DISEASES OF THE UPPER AIR-PASSAGES.*

Among the diseases of the upper air-passages we have first—two varieties, acute nasal catarrh and chronic or naso-pharyngeal catarrh. In the latter subdivision we have the atrophic and hypertrophic varieties and the form which is a mere manifestation of some general systemic disease, *i. e.*, the ozena of syphilitics. The first two varieties are merely different degrees of the same process. The nasal membrane after a series of acute attacks becomes chronically inflamed with a muco-purulent discharge. The membrane is congested and covered with a thin muco-pus. In this form treatment is most effective. But as the disease progresses the membrane becomes hypertrophied and a chronic tenacious mucus is secreted. This hypertrophic form is generally accompanied by a stenosis of the upper air-passages, especially on the convex surfaces of turbinated bones, which eventually become enlarged, and there is a constant dropping of mucus into the back of the throat. Still more chronic is that atrophic catarrh where the mucosa is atrophied and the secretion is dried up.

The treatment of these cases is simple, if seen at the outset. The principle of cleanliness underlies the whole thing. The patient should be supplied with some good alkaline solution, such as glyco-thymoline, and instructed to douche the nasal passages twice daily. This, with proper hygienic managing of the case, is all that is necessary in the majority of the cases, seen at the beginning of their chronicity. If the follicles are hypertrophied they may be touched up with the galvano-cautery. This especially applies to the naso-pharynx.

---

*By Clarence G. Clark, M.D., Assistant Surgeon to Presbyterian Hospital, O. P. D., New York; formerly Assistant Surgeon to New York Hospital, O. P. D.

The best form of douche for use in these cases is the Bermingham. It is easy for the patient to use this douche, and its size makes it convenient to carry. It is a bad practice to use a syringe as too much force may force the discharge into the frontal sinus, and cause unpleasant complications. If the turbinated bones are greatly enlarged, an operation for their removal is necessary before treatment can be effective.

For use in the douche I have found glyco-thymoline to be an excellent preparation. It has the advantage of being alkaline and at the same time it is pleasant to use, and does no harm if swallowed. The following cases will illustrate the treatment of these cases :

Case I. Mr. A. B., age thirty-five. Came to office June 12, 1902, complaining of nasal discharge. History of several acute attacks of ordinary coryza, which gradually became more lasting. Is a heavy smoker and inhales smoke.

Found nasal membrane congested and covered with tenacious mucus. Turbinated bones slightly hypertrophied.

*Diagnosis:* Chronic nasal catarrh, becoming hypertrophic.

*Treatment:* Prescribed—

| | |
|---|---|
| Ac. Tannici | gr. xx. |
| Ac. Sulph. Arom | M. xxx. |
| Glyco-Thymoline | ℥ iii. |
| Aqua dist | q. s. ℥ viii. |

and instructed patient to use same in Bermingham douche twice daily, first cleansing the nasal membrane with a salt solution ʒi to quart of warm water.

As the discharge became less tenacious and less profuse I stopped the astringents and had patient continue with glyco-thymoline in proportion of one part in four of water. With this treatment the discharge ceased in three weeks, and patient was discharged with instructions to continue using the douche daily.

Case II. Miss J., age twenty-four. Came to office July 10, 1903. Has acute coryza, running from nose and eyes. Discharge, thin mucus. Alae nasi excoriated. History of frequent previous attacks.

*Diagnosis :* Acute coryza.

*Treatment:*

| | |
|---|---|
| Atrophin Sulph | gr. 1/10. |
| Pulv. Ipecac et Op | gr. iv. |
| M. et ft pulv. no | viii. |
| Sig.—One every four hours. | |

also instructed the patient to douche every three hours with

| | |
|---|---|
| Glyco-Thymoline | ℥ ii. |
| Aq | q. s. ℥ vi. |

this latter to be used in Bermingham douche.

This attack subsided in ten days, and patient continued using the douche daily thereafter, and up to the present time has had no similar attacks. I lay this to the constant cleansing of the nasal membrane.

